# Prenatal stress unmasks behavioral phenotypes in genetic mouse models of neurodevelopmental disorders

**DOI:** 10.3389/fnbeh.2023.1271225

**Published:** 2023-09-22

**Authors:** Kathryn M. Harper, Samuel J. Harp, Sheryl S. Moy

**Affiliations:** ^1^Department of Psychiatry, University of North Carolina at Chapel Hill School of Medicine, Chapel Hill, NC, United States; ^2^Carolina Institute for Developmental Disabilities, University of North Carolina at Chapel Hill School of Medicine, Chapel Hill, NC, United States

**Keywords:** anxiety, sociability, maternal behavior, memory, ASD, schizophrenia

## Abstract

Neurodevelopmental disorders (NDDs) are complex conditions characterized by heterogeneous clinical profiles and symptoms that arise in infancy and childhood. NDDs are often attributed to a complicated interaction between genetic risk and environmental factors, suggesting a need for preclinical models reflecting the combined impact of heritable susceptibility and environmental effects. A notable advantage of “two-hit” models is the power to reveal underlying vulnerability that may not be detected in studies employing only genetic or environmental alterations. In this review, we summarize existing literature that investigates detrimental interactions between prenatal stress (PNS) and genes associated with NDDs, with a focus on behavioral phenotyping approaches in mouse models. A challenge in determining the overall role of PNS exposure in genetic models is the diversity of approaches for inducing stress, variability in developmental timepoints for exposure, and differences in phenotyping regimens across laboratories. Identification of optimal stress protocols and critical windows for developmental effects would greatly improve the use of PNS in gene × environment mouse models of NDDs.

## 1. Introduction

The prenatal maternal environment plays a key role in brain development of offspring, and conditions with negative effects on the psychological health of the mother can, in turn, have detrimental consequences for unborn children. In particular, clinical studies have suggested that exposure to prenatal maternal stress (PNS) could increase risk for behaviors associated with attention deficit and hyperactivity disorder (ADHD), autism spectrum disorder (ASD), schizophrenia, and other types of neurodevelopmental disorders (NDDs; Kinney et al., 2008b; Manzari et al., 2019). Evidence from rodent models has shown that PNS can alter neurochemistry, neuroendocrinology, and behavior in ways that are consistent with NDD profiles in humans, supporting a link between maternal psychological distress and negative outcomes in offspring (Weinstock, 2017; Haq et al., 2021).

More than a thousand genes have been linked to NDD risk, with findings from human genomic studies available in multiple databases (Leblond et al., 2021). Current understanding supports that most cases of NDDs arise from complex interactions between candidate genes and/or environmental factors (Kim and Leventhal, 2015; Vorstman et al., 2017; Hollander et al., 2020). In line with this premise, mouse models with mutations in a single high-confidence gene are sometimes found to have absent or mild phenotypes related to clinical profiles, raising the possibility that an additional environmental challenge or “hit,” such as PNS, is necessary to drive the emergence of abnormal behaviors (Jiménez et al., 2022; Lord et al., 2022). In this paper, we review the literature examining PNS in genetic mouse models of NDDs, and discuss potential experimental factors that could influence results of these studies.

## 2. Prenatal stress

Studies in humans have examined the effects of stress during pregnancy on the resulting physical and mental health of children. A review of prenatal exposure to maternal stress found that various types of stress were associated with long-lasting behavioral and psychological effects on offspring (Van den Bergh et al., 2020). These effects included changes in motor development, temperament, and emotional regulation. The severity of effects on offspring were dependent on both the magnitude of the stress encountered and the timing during pregnancy when the stressors occurred. A study in central Iran found that maternal stress during months 4–7 of pregnancy significantly increased the risk of ASD (Mousavi et al., 2018). Similarly, a large Swedish population-based study revealed that third-trimester prenatal stress increased the risk of ASD and ADHD (Class et al., 2014). These findings align with previous findings, including a study that found increased ASD rates in children whose mothers were exposed to hurricanes during pregnancy, and another that linked the death of a close relative during the first trimester to increased rates of schizophrenia in offspring (Khashan et al., 2008; Kinney et al., 2008a). In these human studies, it is possible that the prenatal maternal stress induced changes in offspring through epigenetic processes, such as DNA methylation and histone modifications, that led to altered gene expression in the developing brain, with long-term consequences for physiological and psychological function (Babenko et al., 2015; Goyal et al., 2019).

The house mouse (*Mus musculus*) is a very useful genetic model for several human conditions, and it was used in all the papers discussed in this review. Henceforth, when the term mouse is used, it refers to the house mouse. However, one difficulty in comparing the developmental periods of humans and the mouse is the fact that the gestational time periods differ between the species. At birth, a newborn mouse and a newborn human are distinct in their stage of physiological and neural development. According to Parnell et al. (2014), “based on developmental events, approximately half of the human second trimester-equivalent as well as all of the third trimester-equivalent occur after the 19–21 day gestational period in the mouse.” These authors also suggest that the first trimester of human pregnancy is roughly equivalent to day 16 of gestation in mice. This difference in developmental timing suggests that it may be particularly difficult to use mouse models to understand the effects of third-trimester prenatal stress in humans, as the equivalent developmental time period in the mouse occurs postnatally. Some authors have wondered whether other animal models may provide a better model for understanding human pregnancy and gestation, such as the Cairo spiny mouse (*Acomys cahirinus*) that has a longer gestation and has been used for prenatal studies that do not involved genetic manipulations (Carter, 2020). However, as models of PNS in the first and second trimesters of human pregnancy, the house mouse models can provide valuable insights.

Another drawback for mouse models combining genetic liability with PNS is that behavioral outcomes across different laboratories can be inconsistent or even contradictory, likely due to the wide variability in prenatal stress paradigms, including specific stressors used and gestational days of exposure. For example, the studies reviewed in this paper included the following PNS protocols (Table 1):

- Dams were exposed from gestational day (GD) 11.5–GD 17.5 to a schedule of repeated variable stress, including restraint stress, open field exposure, forced swim, cage change, and exposure to unfamiliar females (Oliver and Davies, 2009).
- Starting from GD 5–9 or 8–12 and continuing through the lactation, dams were exposed to soiled bedding from unfamiliar males (Heiming et al., 2009, 2011).
- From GD 13–17, dams were restrained in beakers with water for 45 min, three times daily (Van den Hove et al., 2011; Schmidt et al., 2017, 2022).
- From GD 7–19, dams were given 30 min of restraint twice daily (Heslin and Coutellier, 2018).
- From GD 13–19, dams were given 45 min of restraint three times daily (Clarke et al., 2019).
- Dams were exposed from GD 15–2 to a schedule of repeated variable stress, including restraint stress with or without light, and forced swim (Ben Hamida et al., 2022).
- From GD 12–18, dams were exposed to variable stress, including constant lighting, restraint stress, exposure to fox odor, exposure to novel objects, exposure to novel noise, cage changes, and saturated bedding (Papale et al., 2022).
- From GD 14–21, dams were exposed to a repeating schedule of restraint stress, wet bedding, cage changes, and exposure to novel objects (Petroni et al., 2022a,b).

**Table 1:** Papers using PNS in genetic models of neurodevelopmental disorders. PNS: Prenatal stress. HET: Heterozygous. KO: Knockout. GD: Gestational Day. PD: Postnatal Day.

Reference	Target gene/mouse Line	PNS time period	PNS method	Offspring sex and age at testing	Offspring behavioral assays	PNS × gene phenotypes
Oliver and Davies (2009)	*Snap-25* HET and *Snap-25* Blind-drunk point mutation (Bdr)	GD 11.5-17.5	Variable stress in AM/ PM (restraint/forced swim/open field/cage change/social stress).	Males, 8-11 weeks	Open field Elevated plus maze Y-maze Olfactory 3-chamber choice test Novel object recognition Forced swim Fear conditioning Prepulse inhibition	PNS in Bdr-mutant mice led to increased anxiety in open field and elevated plus maze, and loss of sociability and social novelty preference.
Heiming et al. (2009)	*Slc6a4* (5-HTT) HET and KO	GD 8-12 initiation of PNS; continued into postnatal period	Dams exposed every 2–3 days to soiled bedding from unfamiliar males. Five exposures during gestation and 2-5 during lactation.	Males and females, 7-9 weeks	Elevated plus maze Open field Light/dark	PNS in male and female HET mice increased levels of anxiety-like behavior in the light/dark test and decreased measures of exploratory activity in the open field and light/dark test.
Heiming et al. (2011)	*Slc6a4* (5-HTT) HET and KO	GD 5-9 through PD 15-18	Dams exposed every 2–3 days with soiled bedding from unfamiliar males. Four exposures during gestation and 6 during lactation.	Males and females, 4 weeks	Light/dark Open field Elevated plus maze Free exploration	PNS-exposed HET males had significantly reduced time spent in the free exploration arena, compared to non-exposed HET males. PNS in HET females led to significantly fewer entries into the open arm and reduced time in the open arm of the elevated plus maze, compared to non-exposed HET females.
Van den Hove et al. (2011)	*Slc6a4* (5-HTT) HET	GD 13-17	Chronic restraint in 250mL breaker filled up to 5mm water under bright lights (45min 3x per day)	Males and females, 8 weeks	Novel object Elevated plus maze Forced swim	HET females that were exposed to PNS showed increased depressive-like behavior.
Schmidt et al. (2017)	*Nr3c1* (*Gr)* HET	GD 13-17	Chronic restraint in 250mL breaker filled up to 5mm water under bright lights (45min 3x per day)	Males 24-33 weeks	Ultrasonic communication	No phenotypes
Heslin and Coutellier (2018)	*Npas4* HET	GD 7-19	Chronic restraint stress (30 min 2 x per day).	Males and females, 8-9 weeks	Open field Self-grooming 3-chamber social test Social interaction	PNS in HET males, but not females, led to deficits in social recognition, increased time interacting with an unfamiliar mouse, and reduced time in the center of the open field.
Clarke et al. (2019)	*Nrg1* HET	GD 13-19	Chronic restraint stress (45 min 3 x per day).	Males and females, 7-10 weeks	Prepulse inhibition Light/dark Social interaction Open field Novel object recognition Forced swim Morris water maze	PNS in HET males and females led to hyperactivity in the open field.
Ben Hamida et al. (2022)	*Gpr88* KO	GD 15-21	Unpredictable variable stressors (restraint with or without light, and forced swim)	Males and females, 2 months	Attentional set shifting task	KO exposed to PNS needed more trials to reach criteria and made more wrong trials.
Papale et al. (2022)	*Cntnap2* HET	GD 12-18	Variable daily stressor (constant light, fox odor, novel objects, restraint, noise, cage changes, wet bedding).	Males and females, 3-4 months	Open field Light/dark Elevated plus maze Forced swim 3-chamber social test Social interaction	PNS-exposed HET females had altered social behaviors in the 3-chamber social test and a 10-min reciprocal social interaction test, and increased repetitive behaviors under social conditions.
Petroni et al. (2022a)	*Fmr1* KO	GD 14-21	Unpredictable variable stress in the last week of gestation (alternating days of restraint stress + wet bedding and cage changes).	Males, 3 months, with retest at 18 months	Elevated plus maze Open field Y-maze Social interaction Ultrasonic communication	PNS in older male KO mice led to decreased spontaneous alteration in the Y-maze. PNS-exposed adult male KO mice had lowest levels of social interaction.
Petroni et al. (2022b)	*Fmr1* male KO and female HET	GD 14-21	Unpredictable variable stress in the last week of gestation (alternating days of restraint stress + wet bedding and cage changes).	Males and females, 7-8 weeks	Open field Y-maze Social interaction Ultrasonic communication	PNS in KO males led to decreased spontaneous alteration in the Y-maze.
Schmidt et al. (2022)	*Bdnf* HET	GD 13-17	Chronic restraint in 250mL breaker filled up to 5mm water under bright lights (45min 3x per day)	Males and females, 15-17 weeks	Open field Nest test T-maze	No phenotypes

PNS, prenatal stress; HET, heterozygous; KO, knockout; GD, gestational day; PD, postnatal day.

The differences in PNS paradigms make it hard to draw conclusions across studies, but most studies cover the mouse equivalent of the late first-trimester to the early second-trimester of human pregnancy. Although the type of stress differed across studies, most of the publications listed above reported at least one significant effect of PNS in a genetic NDD model, indicating that the actual stressor used might not be as important as the timing of the PNS. To investigate this hypothesis, future studies could systematically test the same genetic model with various forms of PNS during the same time developmental time periods and compare the results from the different methods.

## 3. Considerations for preclinical study design

Qualitative and quantitative differences in maternal responses can have long-term effects on offspring behavior (Meaney, 2001; Pedersen et al., 2011; Keller et al., 2019; Bridges, 2020; Jiménez and Zylka, 2021). It is therefore important to control variables that can potentially affect the dam’s behavior. For example, alterations in NDD-associated genes can lead to neglectful maternal behavior in dams (Jimenez et al., 2020; Grabrucker et al., 2021). Breeding strategies pairing wild-type (WT) dams and heterozygous (HET) males can help to eliminate the possibility that effects on offspring are due to poor nurturing related to the dam’s genotype. Another factor to consider is that PNS might alter maternal behavior during the neonatal and pre-weaning periods. Most studies compare the weight and the survival of pups in the PNS and control conditions to determine if maternal behavior is affected. Heiming et al. (2011) showed a decrease in maternal care, but no differences in weight, litter size, or sex ratio between the conditions. Petroni et al. showed a decrease in nursing postures in the dams (Petroni et al., 2022a), but using the same paradigm also found an increase in WT offspring (both males and females) and KO female offspring (Petroni et al., 2022b). Clarke et al. (2019) showed a decrease in nursing, but overall positive effects of PNS on offspring behavior. These data suggest that weights are not a good indicator of maternal behavior. However, these results do confirm that offspring can still have a healthy development despite deficits in maternal behavior. Taking periodic measures of pup weights can help detect issues related with deficient maternal care, as well as direct effects of PNS and/or genotype on offspring. Cross-fostering can be used to control for stress effects on maternal behavior. However, cross-fostering can create its own negative effects on offspring behavior and requires twice as much breeding (McCarty, 2017).

An additional concern is that individual variation in a dam’s response to stress can lead to litter effects, in which measures taken from pups within a specific litter correlate. One way to control for litter effects is by using only one to three offspring per litter for a particular study (Schmidt et al., 2017, 2022; Heslin and Coutellier, 2018; Papale et al., 2022). Alternatively, statistical methods are available that control for litter in the analyses (Jiménez and Zylka, 2021).

Finally, both sexes should be used in any future studies, as many studies find the effects of PNS to be different in males and females. The endocrine system is thought to play a role in the effects of PNS (de Souza et al., 2013; McGowan and Matthews, 2018), which could explain the differences between the sexes. Additionally, sex differences in vulnerability to PNS could reflect similar differences in NDDs in the human population.

## 4. Use of PNS in mouse models of NDD genetic susceptibility

The generation of a mouse knockout line is often the first step in developing a genetic mouse model. In cases where the full deletion is lethal, as with some high-confidence NDD genes (Oliver and Davies, 2009; Schmidt et al., 2017, 2022; Clarke et al., 2019; Jimenez et al., 2020), the heterozygous mutant can serve as a potential model. Notably, the HET genotype can be more reflective of mutant alleles in human disorders than null deletions (Toma et al., 2014; Michetti et al., 2022), with the drawback that partial loss of a gene does not have the same impact as total knockout. For example, our group found that heterozygosity is insufficient to lead to overt ASD-like phenotypes in mice with partial loss of *Chd8*, a high-confidence ASD gene (Jimenez et al., 2020). Similarly, *Shank3* mutant mice have significant behavioral changes with homozygous, but not heterozygous, loss of function (Lord et al., 2022). These models with mild phenotypes are promising candidates for further investigation using PNS, since stronger abnormalities might lead to floor or ceiling effects, in which further alteration of behavior would be difficult.

Statistical analysis of studies combining genetic vulnerability with PNS exposure can be complex, especially with the additional factor of sex and the use of repeated measures. One strategy is to first conduct overall comparisons to determine main effects and interactions for genotype, stress condition, and sex, and then carry out separate analyses for males and females to identify within-sex genotype and stress condition effects. Significant main effects or interactions could then be further explored with planned comparisons between genotype groups within each stress condition. These final comparisons could indicate the presence of genotype differences in the PNS groups, but not the non-stressed groups, in line with an underlying genetic liability driven by an environmental exposure.

## 5. Overview of published studies: PNS effects in genetic mouse models of NDDs

Papers for this review were found by searching Google Scholar and PubMed for the terms “prenatal stress” and “mice” in combination with one of the following terms: autism, schizophrenia, or neurodevelopmental disorders. Papers that lacked behavioral analysis or focused on the genotype of the dam rather than the genotypes of the offspring were excluded. The search revealed twelve papers investigating PNS in genetic mouse models of NDDs (Table 1) that employed a range of behavioral tests, including assays for social approach, anxiety-like behavior, repetitive responses, and learning and memory. Nine genes were targeted in the models:

- *Bdnf* (Brain derived neurotrophic factor), a gene for a neurotrophic factor involved in neuronal survival and neurogenesis. Altered levels of *Bdnf* have been found in ASD and schizophrenia (Schmidt et al., 2022).
- *Cntnap2* (Contactin associated protein 2), a gene for a neuronal cell-adhesion protein, associated with Pitt-Hopkins syndrome and ASD (Papale et al., 2022).
- *Fmr1* (Fragile X messenger ribonucleoprotein 1), a gene located on the X chromosome, associated with fragile X syndrome, intellectual disabilities, and ASD (Petroni et al., 2022a,b).
- *Gpr88* (G-protein coupled receptor 88), encodes an orphan G protein coupled receptor, associated with schizophrenia and ADHD (Ben Hamida et al., 2022).
- *Nr3c1* (nuclear receptor subfamily 3, group C, member 1), a gene for the receptor that binds glucocorticoids, leading to regulation of gene transcription and associated with ADHD (Schmidt et al., 2017).
- *Npas4* (Neuronal PAS domain protein 4), a gene for a transcription factor with a role in excitatory/inhibitory balance, associated with ASD, schizophrenia, and other NDDs (Heslin and Coutellier, 2018).
- *Nrg1* (Neuregulin 1), a gene for a neurotrophic factor involved in synapse formation and synaptic plasticity, associated with schizophrenia (Clarke et al., 2019).
- *Slc6a4* (Solute carrier family 6 member 4; 5-HTT), encodes the serotonin transporter, which is involved in serotonin reuptake; associated with anxiety disorders (Heiming et al., 2009, 2011; Van den Hove et al., 2011).
- *Snap-25* (Synaptosomal-associated protein, 25 kDa), a gene for a protein important for exocytosis in neurons, associated with ADHD and schizophrenia (Oliver and Davies, 2009).

### 5.1. Social approach and interaction

The 3-chamber choice test for sociability and social novelty preference was used in three of the reviewed studies. In this test, mice were presented with an unfamiliar mouse enclosed in a small cage, which allowed limited interaction without aggression, chasing, or other active interactions. The method for the test differed across the papers, and the “stranger” mice used varied by sex, strain, and stage of development (i.e., juvenile or adult). Notably, genotype differences were only observed in the groups exposed to PNS. In particular, female *Cntnap2* HET mice (Papale et al., 2022) and male mice with the blind drunk (Bdr) point mutation in *Snap-25* (Oliver and Davies, 2009) that had PNS exposure demonstrated a lack of social preference in the 3-chamber test, while the no-stress groups and WT PNS mice all showed positive sociability. In a subsequent social novelty phase of the task, when presented with a second stranger mouse, the *Snap-25* Bdr mutant mice exposed to PNS were the only group that did not demonstrate a shift in preference to the newly-introduced stranger. Selective deficits in social novelty preference were reported in male *Npas4* HET mice exposed to PNS, in comparison to the other experimental groups (Heslin and Coutellier, 2018).

Five of the studies conducted a direct social interaction test, where the test mouse and the partner mouse were allowed to freely engage. Similar to the results from the 3-chamber task, female *Cntnap2* HET mice in the PNS group, but not the no-stress group, had reduced social interaction time (Papale et al., 2022). An opposite change was observed in male *Npas4* HT mice exposed to PNS, which spent more time than no-stress HET mice sniffing a stimulus mouse (Heslin and Coutellier, 2018). Increased social interactions were also observed in both WT and *Nrg1* HET mice in a direct social interaction test, indicating a general effect of PNS on affiliative behavior (Clarke et al., 2019). In the mouse model of fragile X syndrome, the effects of PNS on direct social interaction varied across genotype and age at testing (Petroni et al., 2022a,b). At 2 months of age, PNS led to increased affiliative behavior in both male and female WT mice, but not *Fmr1* KO males or HET females, perhaps due to overall higher duration of social interaction in the fragile X model (Petroni et al., 2022b). In contrast, a separate study found that the same PNS regimen failed to have significant effects on affiliation in male mice at 3 months in age, although the data suggest a trend for lower interaction time in the *Fmr1* KO mice exposed to PNS (Petroni et al., 2022a).

Overall, PNS was associated with decreased social preference in the 3-chamber test, but both decreased and increased affiliative behavior in tests of direct social interaction, dependent upon the particular model and age of testing. While reduced sociability could reflect the social deficits characteristic of ASD or schizophrenia, increased social preference, or hypersociability, could be relevant to other aspects of NDD clinical profiles, such as risky, impulsive behavior and impaired inhibitory control (Vara et al., 2014; Velasquez et al., 2017; Mirabella, 2021).

Heiming et al. (2009, 2011), Van den Hove et al. (2011), Schmidt et al. (2017, 2022), and Ben Hamida et al. (2022) did not include social testing in their studies.

### 5.2. Anxiety-like behavior

Anxiety is a common co-morbidity in NDDs, and tests for anxiety-like behavior are often included in phenotyping regimens for NDD models. Ten of the reviewed papers employed at least one standard assay for anxiety-like behavior, including the elevated plus maze (EPM) or zero maze, open field (OF), or light/dark preference test (LD).

PNS had no significant effects in *Bdnf* HET mice tested in an open field (Schmidt et al., 2022).

The results in ten reports showed wide variability in the effects of PNS, both across and within models. For example, no effects of PNS or genotype were found in *Cntnap2* HET mice in the EPM, OF, or LD tests (Papale et al., 2022), or *Nrg1* HET mice for time spent in the “hide box” during a LD test (Clarke et al., 2019). Similarly, PNS had no significant effects on time in center in *Bdnf* HET mice tested in an OF (Schmidt et al., 2022). On the other hand, PNS led to general increases in anxiety-like behavior in WT and *Brd* mutants tested in the EPM and OF (Oliver and Davies, 2009), and in 3-month-old WT and *Fmr1* KO mice (Petroni et al., 2022b). In the *Npas4* HET model, a significant gene x stress condition interaction was found for time spent in the center of the OF; further analyses revealed that male, but not female, *Npas4* HET mice exposed to PNS had less center time than the non-exposed HET males, without any concomitant changes in general locomotor activity (Heslin and Coutellier, 2018).

PNS also had variable effects on anxiety-like behavior in *Slc6a4* HET and KO mice. Heiming et al. (2009) reported that in the LD test, *Slc6a4* KO mice exposed to PNS, but not the no-stress group, had longer latencies to enter light compartment than other experimental groups. PNS also led to a decrease in entries into the light compartment in WT mice; notably, the KO mice in both stress conditions had fewer entries into the light compartment than WT controls. In contrast, no significant effects of genotype or stress condition were found for percent time spent on the open arms of the EPM, or percent center time in the OF (Heiming et al., 2009). The significant PNS effects in the LD test were not replicated in a second study with the same model (Heiming et al., 2011); further, significant differences between stress conditions were limited to *Slc6a4* HET mice, versus mice carrying the null deletion. In this case, *Slc6a4* HET females exposed to PNS had lower percent time on and entries into the open arms of the EPM than HET females in the no-stress group. Another paper investigating the same model found that *Slc6a4* HET mice had general increases in percent time on the open arms of the EPM, independent of stress condition, while the main impact of PNS was a general decrease in distance traveled on the maze (Van den Hove et al., 2011). Overall, these findings indicate that PNS effects in a single genetic model can be inconsistent across tests and studies, even within the same laboratory.

Schmidt et al. (2017) and Ben Hamida et al. (2022) did not include tests for anxiety-like behaviors in their studies.

### 5.3. Repetitive behavior

Repetitive behavior is a common feature of NDDs in humans (Whitehouse and Lewis, 2015); however, only two of the reviewed papers evaluated mice for repetitive responses (grooming and digging). Papale et al. (2022) found that WT and *Cntnap2* HET mice had comparable levels of grooming/digging when tested in a cage alone. In contrast, when placed with an unfamiliar stranger, the *Cntnap2* HET mice exposed to PNS had higher levels of grooming/digging than any other group, suggesting that the early PNS exacerbated sensitivity to a stressful social situation, leading to the emergence of repetitive behavior in the *Cntnap2* model. An opposite effect of PNS was observed in male *Npas4* HET mice, in which the exposed group had lower levels of grooming than the no-stress HET group; this significant effect of PNS was not observed in the WT mice, or in the female HET groups (Heslin and Coutellier, 2018).

Heiming et al. (2009, 2011), Oliver and Davies (2009), Schmidt et al. (2017, 2022), Petroni et al. (2022a,b), and Ben Hamida et al. (2022) did not include a test of repetitive behavior.

### 5.4. Learning and memory

Five of the reviewed papers used novel object recognition or Y-maze alternation tests to evaluate learning and memory. In *Slc6a4* HET mice, the exposure to PNS led to general deficits in novel object recognition in the female WT and HET groups after a 2-h delay from presentation of the objects (Van den Hove et al., 2011). Interestingly, after a 3-h delay, an overall effect of genotype emerged, in which the male and female HET mice had better memory performance than WT, independent of stress condition. No significant effects of either PNS or genotype were found for novel object recognition in *Nrg1* HT mice (Clarke et al., 2019) or *Snap*-25 HET or *Bdr* mutant mice (Oliver and Davies, 2009). Further, the *Snap-25* models had performance comparable to WT in the Y-maze and fear conditioning tests, independent of stress condition (Oliver and Davies, 2009). A different pattern was observed in the *Fmr1* KO model, in which all of the groups (male no-stress and stress WT, and no-stress *Fmr1* KO), except the KO mice exposed to PNS, demonstrated significant alteration in the Y-maze, dependent on age at testing (Petroni et al., 2022a,b). The T-maze, which can be used similarly to the Y-maze, showed only a decrease time spent in the new arm in *Bdnf* HET mice from both sexes and stress conditions, indicating only genotype had a negative effect on cognitive performance (Schmidt et al., 2022).

Only one study (Clarke et al., 2019) included a hidden platform test in the Morris water maze, a widely-used procedure for evaluating spatial learning in mice. In this case, *Nrg1* HET mice exposed to PNS had *better* performance than the no-stress HET group (Clarke et al., 2019). Finally, Ben Hamida et al. (2022) used the attentional set-shift task, which uses a series of discrimination learning tasks to identify deficits in attention, relevant to ADHD. The researchers found that *Gpr88* KO mice required more trials to reach criteria and had more incorrect trials than the WT controls, and these deficits were exacerbated by PNS exposure.

No test of learning and memory was included in Heiming et al. (2009, 2011), Schmidt et al. (2017), Heslin and Coutellier (2018), or Papale et al. (2022).

## 6. Conclusion

**Figure 1:**
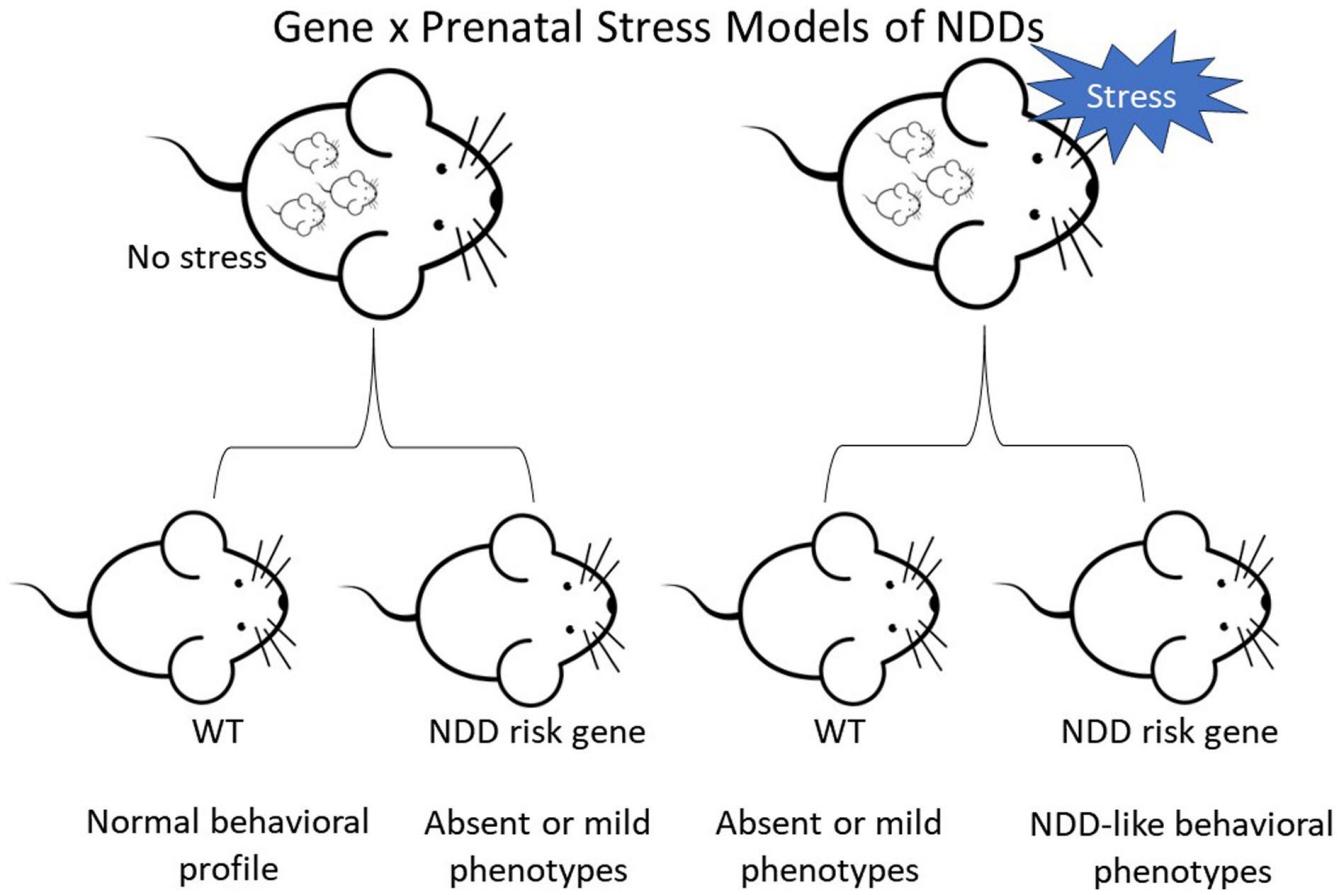
Diagram depicting the two-hit hypothesis of neurodevelopmental disorders (NDDs) in mice. Top row depicts pregnant female mice that are either controls or undergo stress. Offspring are wild-type (WT) or carriers of a NDD risk gene.

In preclinical mouse models, the alteration of genes identified as high-confidence risk factors in NDDs does not always result in robust phenotypes reflecting symptoms in the human disorders. However, mutant lines with mild or absent behavioral changes provide an opportunity to investigate the impact of environmental factors on heritable vulnerability. The papers summarized above indicate that PNS exposure can interact with underlying genetic susceptibility, leading to the emergence of abnormal phenotypes in NDD mouse models (Figure 1). At the same time, our overview shows that the impact of PNS is not consistent across NDD mouse models, PNS protocols, or domains of behavioral function. For example, PNS exposure had divergent general and genotype-specific effects on anxiety-like behavior, with wide variability even in studies using the same genetic model and PNS protocol. More consistency is needed in PNS paradigms and behavioral test parameters in the future, but additional candidate NDD genetic models with milder phenotypes or only a few phenotypes should be tested.

Interestingly, selective effects of PNS exposure were observed in *Cntnap2* HET mice across tests for social approach and interaction (Papale et al., 2022). The genotype-dependent PNS effects included increased repetitive behavior in the HET group when evaluated a social setting. Notably, *CNTNAP2* is a high-confidence gene for risk of ASD, a set of disorders with hallmark features of social deficits and abnormal repetitive behavior. The findings from Papale et al. (2022) provide validation for the *Cntnap2* HET mouse as a model for gene × environment interactions in ASD. Similarly, Ben Hamida et al. (2022) reported that PNS exposure worsened attention deficits in *Gpr88* KO mice, a proposed model for ADHD. The researchers also showed that, in a clinical study, including maternal stress and obstetrical complications as a factors in genetic analysis strengthened the association of a specific *GPR88* variant with risk for ADHD. Overall, these findings support future work on the optimization of PNS protocols for “two-hit” genetic models of ASD and other NDDs.

## Author contributions

KH: Writing – original draft, Writing – review & editing. SH: Writing – original draft, Writing – review & editing. SM: Writing – original draft, Writing – review & editing.

## Funding

The author(s) declare financial support was received for the research, authorship, and/or publication of this article. This work was supported by a grant from the Eunice Kennedy Shriver National Institute of Child Health and Human Development (NICHD; P50 HD103573; PI Gabriel Dichter).

## Conflict of interest

The authors declare that the research was conducted in the absence of any commercial or financial relationships that could be construed as a potential conflict of interest.

## Publisher’s note

All claims expressed in this article are solely those of the authors and do not necessarily represent those of their affiliated organizations, or those of the publisher, the editors and the reviewers. Any product that may be evaluated in this article, or claim that may be made by its manufacturer, is not guaranteed or endorsed by the publisher.
